# A Case of Gas Gangrene Associated with B. OEdematiens

**Published:** 1917-12

**Authors:** 


**A Case of Gas Gangrene Associated with B. OEdematiens.** By E. J. Dalyell, of the Bacteriological Laboratory, Hôpital Auxiliaire 301 (Scottish Women's Hospital), France. Abs. from the *British Medical Journal*, March 17, 1917.

D. describes in full experiments made with bacilli isolated from a severe case of gaseous gangrene that proved fatal. The author subjected it to practically the same tests as those described by Weinberg and Séguin. He states : " In all the features of morphology, cultural character, auto-agglutination, and serum tests, the organism conformed to the description given by Weinberg and Seguin of B. œdematiens. (Dr. Weinberg confirmed the identification).

The author notes the great difficulty involved in isolating the organism. Strict anaerobiosis is essential, and this can be obtained only by the method employed at the Pasteur institute, which the author describes as follows :

" The test tube containing fluid medium is inoculated and is then heated above the level of the fluid and drawn out into a thin neck. The mouth of the tube is unaltered, and the cotton-wool plug is pushed down towards the constriction and the tube attached to a pump and exhausted. During the process of exhaustion gentle heating of the fluid drives out all air dissolved in the liquid, and the tube is then sealed by Bunsen flame at the constricted neck, and is ready for incubation. "

The author adds that B. œdematiens is associated with a peculiarly severe form of gas gangrene, characterized by an acute general intoxication and extensive spreading solid edema, with little gas formation other than that due to associated organisms in the neighborhood of the wound. He believes that a more careful search under anaerobic conditions would reveal its presence in many cases of gas gangrene.

